# Stylistic analysis of translated languages: A perturbation-based XAI deep learning framework

**DOI:** 10.1371/journal.pone.0352889

**Published:** 2026-07-07

**Authors:** Dan Feng Huang, Dennis Tay

**Affiliations:** 1 School of Foreign Languages, Guangdong Polytechnic Normal University, Guangdong, China; 2 Divison of Humanities, The Hong Kong University of Science and Technology, Hong Kong SAR, China; Ariel University, UNITED KINGDOM OF GREAT BRITAIN AND NORTHERN IRELAND

## Abstract

Text classification using traditional machine learning techniques has been used in natural language processing (NLP) tasks to distinguish translated from non-translated languages, with high accuracy scores indicating the distinctive style of translated languages. While deep learning (DL) has demonstrated impressive performance in terms of representation learning and capturing nuanced patterns in natural language data, DL models act as black boxes, making their results difficult to interpret. This study addresses this issue by demonstrating an explainable AI (XAI) DL framework in a case study of United Nations (UN) meetings. The framework consists of three stages: i) train a variational autoencoder (VAE) combined with BERT embeddings converted from translated and non-translated texts; ii) utilize the majority vote from three classifiers selected from a stacked ensemble to classify the VAE’s latent representations; iii) implement a perturbation-based XAI method to interpret the DL model’s decisions. The results indicate that the VAE-based model effectively distinguishes the two text types, with accuracy scores above 0.8. The XAI analysis reveals that interpreting the VAE-based model’s decision uncovers stylistic differences between the two text types beyond superficial lexical and syntactic features. This proof-of-concept study demonstrates the potential of the XAI DL framework in other NLP studies that aim to analyze style.

## Introduction

**Fig 1:**
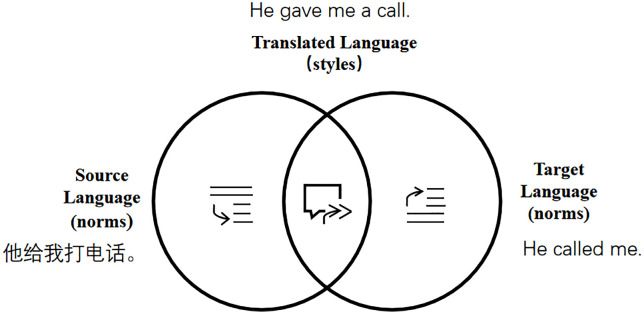
Source, target and translated languages.

Translation, which is used in a broad sense in this study to include both written and oral translation, is a type of mediated cross-cultural communication between source and target languages [1,2]. Due to the source language interference, translated languages often exhibit distinctive linguistic features from the target language [3,4]. For example, as shown in Fig 1, the English translation “He gave me a call” is grammatically correct and understandable. However, influenced by the Chinese source sentence “他给我打电话” (“给” for “gave”), the translated sentence differs from the more idiomatic English expression “He called me”. Many such usages contribute to a distinctive style inherent in translated languages, which cannot be explained by grammar or word choice alone. Accurately capturing this style would help us to understand why translated languages sound unnatural and enhance the chances of the translated outputs being acceptable to stakeholders.

A new trend of studies has employed text classification for this distinctive style detection [5–16]. Text classification is a supervised machine learning (ML) technique to assign items to predefined categories by modeling the relationship between input features and known labels [17]. In these studies, the metrics for linguistic features were extracted and used to train classification models to distinguish translated texts from non-translated texts in the target language (referred to as “non-translated texts” in short hereinafter). The higher the classification accuracy scores, the better the input features are at predicting the two text types, which further suggests that the two text types exhibit different linguistic features.

Despite the effective integration of state-of-the-art ML techniques with translation studies, the aforementioned studies have methodological limitations. One group of studies isolated features, often encompassing syntactic complexity [18], lexical sophistication [15], or both [7]. Using isolated features risks cherry-picking in investigations [5,9]. By contrast, a second group of studies relied on word-form and/or part-of-speech n-gram models to capture textual features [6,8,11,12]. Although n-gram models reduce cherry-picking to some extent, they are more effective with local statistical patterns than with deeper contextual or semantic information, which limits their capability to decode style as a feature beyond surface word choices [19,20].

Contemporary deep learning (DL) models offer a promising way to overcome these methodological limitations. DL is a subset of ML utilizing neural networks [21,22]. As an application of DL, pretrained language models like BERT [23] provide deep representations for natural language processing (NLP) tasks [24]. In addition, among the diverse neural networks, the autoencoder families are known for their representation learning in the latent space [25–27]. In their representation learning, salient features are automatically extracted from raw data, as high-dimensional inputs are mapped to compact, lower-dimensional latent representations that retain the essential information necessary for effective reconstruction.

Despite their impressive performance, the DL models face a significant challenge. They are often described as “black boxes” because their internal decision-making processes are complex and not easily explained by humans. Predictions alone are insufficient for stakeholders to gain insight into mechanisms [28,29]. To gain explanations of the phenomena under scrutiny, researchers have attempted various methods, leading to the growing field of explainable artificial intelligence (XAI) [30–32]. Given the many factors that influence model behavior, including data collection practices, network architecture, and downstream tasks, no consensus has been reached on the best approach. Among the flourishing XAI methods, the perturbation method is one of them used to shed light on the black box [33,34]. Perturbation means making a change to the input for the DL model, connecting the input change with the potential change in the DL’s behavior as the output, and thereby providing explanations for the DL model’s decision-making process.

In this proof-of-concept study, we aim to leverage a variational autoencoder (VAE) [35,36] and perturbation-based XAI to explore the distinctive styles of translated languages and propose an XAI DL framework consisting of three phases: i) train a BERT-based VAE to reconstruct translated texts and their comparable non-translated texts; ii) classify the two types of texts using latent vectors and a majority vote based on a stacked ensemble; iii) use perturbation-based XAI to explain the classification results. We apply the framework to categorize translated and non-translated languages used at United Nations (UN) meetings, where simultaneous interpreting, an oral form of translation activity, is conducted. Due to the homogeneity in themes, registers, and speech procedures, UN corpora have been frequently employed in previous studies to investigate how the translation process shapes translated languages [15,37–40]. In this study, the interpreted speeches and their non-interpreted counterparts in the target languages are collected at UN meetings and transcribed to constitute translated and non-translated texts for our case study.

We further elaborate on our framework in Sect 2, in which VAEs and the perturbation XAI are introduced. Sect 3 presents methodological details on corpus construction, the architecture and training of the VAE, the classification scheme, and how the perturbation-based XAI was conducted. Sect 4 presents the results and discussion, and Sect 5 discusses the limitations of the study and offers suggestions for future research.

## The XAI DL framework

### Phase 1: VAE training

In this phase, a VAE was trained with BERT embeddings of the two text types. Style in NLP is believed to emerge from various and random word choices, syntactic patterns, and discourse organizations to demonstrate itself as an abstract and global attribute, such as tone, sentiment, and degree of formality [41–43]. We proposed that through representing input as a smooth and continuous probabilistic distribution within the latent space, VAEs transform stochastic linguistic choices into more stable and organized stylistic features. A more detailed explanation of VAEs’ probabilistic representation learning was provided below.

**Fig 2:**
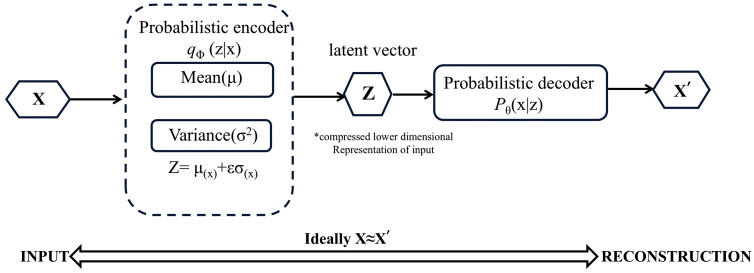
The VAE architecture.

Fig 2 illustrates the architecture of VAEs. As a variant of autoencoder models, a VAE has the basic encoder and decoder structure: It receives input data x from the encoder, completes its learning in the latent space, and through the decoder, reproduces data x’ on the basis of what it has learnt. The close approximation of x to x’ (x ≈ x’) indicates effective representation learning occurring in the latent space.

Different from traditional autoencoders, VAEs capture key representations through probabilistic distribution, which is achieved within a unified probabilistic framework, as illustrated below.

(i) A “reparameterization trick” to convert input as probabilistic distribution (z=μ(x)+σ(x)⊙ε,ε~N(0,I)) [36,44]: the encoder transforms input vectors into two parameters as mean μ(x) and standard deviation εσ(x), which define the approximate posterior $q_{\phi }(z|x)$, transforming input as a Gaussian distribution.
(ii) Variational inference [35,36] for approximation: this process facilitates the learning of effective latent representations by approximating the true posterior distribution $p_{\theta }(z|x)\;$by a learned, tractable distribution $q_{\phi }(z|x)$ provided by the encoder.
(iii) Regulating loss function with Kullback-Leibler (KL) Divergence: KL Divergence measures the difference between the encoder’s approximate posterior $q_{\phi }(z|x)$ and the prior distribution p(z), penalizing the model when the learned latent distributions deviate from the prior, and thereby enhancing VAEs’ probabilistic and approximation representation learning.

Through this unified probabilistic framework, VAEs model input data as a region of possibility within the latent space, capturing the inherent variability and randomness of linguistic usage and encoding the random use as stable and structured stylistic properties. A real-life scenario may facilitate our understanding: In typical school practice, a teacher often teaches two classes at different proficiency levels. Instead of evaluating each student’ s performance, teachers often use summary statistics, such as mean and standard deviation, to represent each class’s academic achievement. While students’ performance (concrete word and expression usages) varies, a teacher (the VAE) could tell whether the class is at an advanced level or an intermediate level by judging from the means and standard deviation scores, with proficiency levels underlying the observable and varied performance of individual students.

In this study, we defined the style of translated languages as a feature that originates with arbitrarily used words and sentence patterns [13,39] but extends beyond the concrete linguistic choices to encompass more global elements, such as sentiment, rhetoric, tone, and underlying cultural nuance [16] and proposed using VAEs’ representation learning to capture the style.

### Phase 2: Majority vote based on a stacked ensemble for classification

In this phase, text classification was performed between the translated and non-translated texts. Considering the principled differences of different classifiers, we implemented a majority vote scheme based on a stacked ensemble framework [45,46]. First, three classifiers, whose combination produced the best classification performance, were identified through a stacked ensemble. To do this, we designed a two-level hierarchical stacked architecture: Level-0 (the base classifiers) and Level-1 (the meta-learner). Five diverse and widely used ML algorithms were selected as base learners for the stack, and one additional classifier was used as the meta-learner to aggregate predictions and make the final classification decision. The optimal set of three classifiers was determined on the basis of the final decision provided by the meta-learner. Subsequently, the selected three classifiers participated in a majority vote to determine the final classification outcomes.

### Phase 3: Perturbation-based XAI

In this phase, we aimed to understand the DL model’s decisions to interpret the stylistic differences between translated and non-translated languages through XAI.

There exists a plethora of XAI methods, which could be primarily categorized according to their scope (local or global), stage (intrinsic or post hoc), methodological approach (perturbation- or attribution-based), and model dependency (model-agnostic or model-specific) [30–32]. For example, Gini Importance is considered an “intrinsically interpretable method” [47] and a model-specific XAI method to find out what a tree-based model considers important features. A local interpretable model-agnostic explanation (LIME), in contrast, is model-agnostic but explains a single prediction rather than providing a global explanation of the model’s behavior.

The perturbation-based XAI [33,34] in this study was motivated by perturbation’s distinct advantages in explaining black-box models such as deep neural networks and large language models (LLMs) [33]. The core idea of perturbation-based XAI is to systematically change the input data and observe how these changes impact a black-box model’s output to explain the model’s decisions. The input-output observation is considered “natural and intuitive” [33]. In addition, perturbation-based XAI is model-agnostic and flexible. It works regardless of the model’s internal structure [48], which makes it ideal for broad application landscapes including perceptual data (images, audio) [49], natural language [50], and code [51]. For example, to investigate the influence of each input word on the behavior of LLMs, Dhar and Devi [50] masked each word and measured the resulting change in output, so perturbation in this study was used as a systematic probing method to provide an interpretable account of LLM’s decision-making. In Manttari, Broomé [49], perturbation was used to identify and alter the most influential frames in a video sequence to reveal what each video model focused on over time for its classification decisions. Instead of masking a word, they masked temporal frames one at a time.

**Fig 3:**
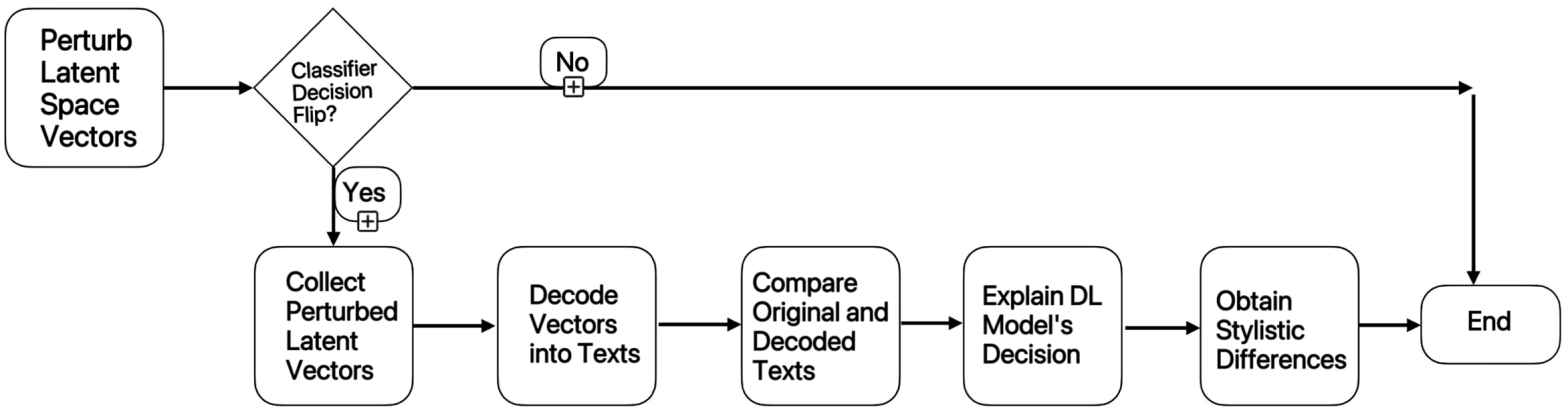
The perturbation-based XAI.

The target of our perturbation intervention was the dimensions of the latent space vectors. Therefore, different from the above-mentioned studies which altered an input outside a DL model, we altered an input in the DL model’s latent space. Consistent with these previous studies, we adhered to the intuitive input-output principle. In other words, the change in output, which referred to the flip of the classification decisions, triggered our interpretations of the input. To do this, when we observed a flip of the classification decision, we collected the perturbed latent vectors and depended on the decoder to obtain the decoded embeddings, which were subsequently converted to text to compare with the original text on the side of the encoder. Through the comparison, and since the two texts were labeled differently by the DL model, we obtained insight into the potential stylistic differences between the two text types. The procedures of the perturbation-based XAI are visualized in Fig 3.

Notably, rather than training a text generation model to convert the decoded embeddings into texts, we depended on cosine similarity calculation [52] to select a text from the whole dataset as the “generated text”. To do this, we matched the decoded embeddings with the embeddings of every text from the whole dataset to find out the best match based on the highest cosine similarity. The best-matched text was then taken as the decoded text (retrieved text). We acknowledge that this is a limitation of our study as the retrieved text was not generated from the latent vectors in a true generative sense but rather retrieved on the basis of similarity. This was a compromise we made due to limited resources and time and the fact that our corpus was not sufficiently large to train a robust text generation model. However, the cosine similarity search method has its merit in ensuring that the retrieved texts remain within the distribution of natural human language. avoiding the risk of generating nonsensical or ungrammatical strings that a poorly trained generator might produce, which could lead to misleading interpretations.

## Research design

### Corpus

Video recordings were downloaded from the official UN website (https://media.un.org/en/webtv/) and transcribed. The non-translated texts referred to the original English speeches delivered by American delegates, and the corresponding translated texts referred to English renditions translated from Chinese. Multiple speakers and interpreters were involved to minimize attribution of style to a single individual’s linguistic preferences, while standardized and conventional linguistic usages required by the meetings reduce confounds from speaker identity. In total, the UN corpus comprises two sub-corpora. More details are available in Table 1.

**Table 1:** The UN corpus.

Corpus	Tokens	Range (years)	Texts
Non-translated English	311, 232	2021−2025	450
Translated English	287,663	2021−2025	450

### Text preprocessing and BERT embedding

Text preprocessing in the study included removing possessive endings, typographic apostrophes, web URLs, and digits; normalizing whitespace; expanding common contractions; converting all words to lowercase; and masking named entities. To further mitigate topical confounds present in the comparable corpus, we used stop words identified through Latent Dirichlet Allocation (LDA) [53] to filter out signals associated with specific topics.

The processed texts were then split into segments, each containing approximately 256 words. For each segment, a 768-dimensional embedding was generated using the all-mpnet-base-v2 Sentence-BERT model [54]. Subsequently, mean pooling was applied across all segment embeddings for a single text, resulting in a single aggregated 768-dimensional vector representing the entire text. The dataset was ultimately split into training (80%) and testing (20%) sets, using a random seed of 42, and features were scaled before being used as input for the VAE’s training.

### The VAE’s architecture and training

Regarding the VAE’s architecture, we designed a symmetric VAE constructed as follows: the encoder comprised three linear layers (with feature dimensions 768 → 512 → 256 → 128), each followed by batch normalization, Leaky ReLU activations (negative slope = 0.2), and a dropout layer (p = 0.1) after the first layer to mitigate overfitting. The encoder produced both the mean and log-variance vectors for the latent code (latent dimension = 50). The decoder mirrored the encoder’s structure, mapping the sampled latent vector back to the input dimension through successive batch-normalized Leaky ReLU-activated layers [55].

For training, we used a reconstruction loss (Mean Squared Error) and a KL divergence loss. The KL term was scaled by β = 1.0 and further modulated via an annealing parameter (kl_anneal), which was increased early in training to improve latent structure. A free bits threshold (0.0–0.5) was applied on the KL divergence to avoid posterior collapse by limiting excessive penalization of individual latent dimensions. The model was trained using Adam [56] (batch size 64, 50 epochs), and training dynamics were monitored by plotting the total loss and KL divergence across epochs.

### Majority vote based on the stacked ensemble

For the stacked ensemble, five base learners were a support vector machine, a random forest, XGBoost, k-nearest neighbors, and a decision tree. Each base learner was configured with pre-optimized hyperparameters to ensure robust and effective learning. To mitigate the risk of data leakage, the meta-learner was trained on out-of-fold predictions generated through a 5-fold cross-validation. Specifically, for each fold, the base models were trained on the remaining k-1 folds and produced predictions for the held-out fold, and these out-of-fold predictions were aggregated to form the meta-learner’s training data. Logistic regression was selected as the meta-learner, tasked with learning weights that optimally combine the predictions from Level-0 models to yield the final classification.

To identify the most effective three-base-learner ensemble, all possible combinations of three classifiers selected from a pool of five were generated using an itertools. combinations function, resulting in (53)=10 unique ensembles. Each ensemble was instantiated as a stacking classifier, trained on scaled training data, and evaluated using the scaled test data. Ultimately, classification accuracy served as the primary metric for performance comparisons. Using this systematic approach, we identified the top-performing three models in the ensemble and used their majority-vote outcomes as the final classification results.

### Iterative perturbations with a minimal epsilon

During this phase, each test sample underwent an iterative process of applying minimal perturbations within the 50-dimensional latent space. Applying minimal perturbations was inspired by adversarial training techniques used in computer vision [57,58] and NLP [59]. In these fields, models are evaluated or enhanced for robustness by being exposed to imperceptible or minimal adversarial attacks, which refer to tiny input changes that may “fool” the models to change their decisions. While there is debate regarding the relevance of imperceptible perturbations [60], the primary objective in previous work was to increase model robustness. In contrast, our approach adopted minimal perturbations with the aim of providing an explanatory basis for the learned representations. Consequently, we maintained a minimum-perturbation principle.

**Fig 4:**
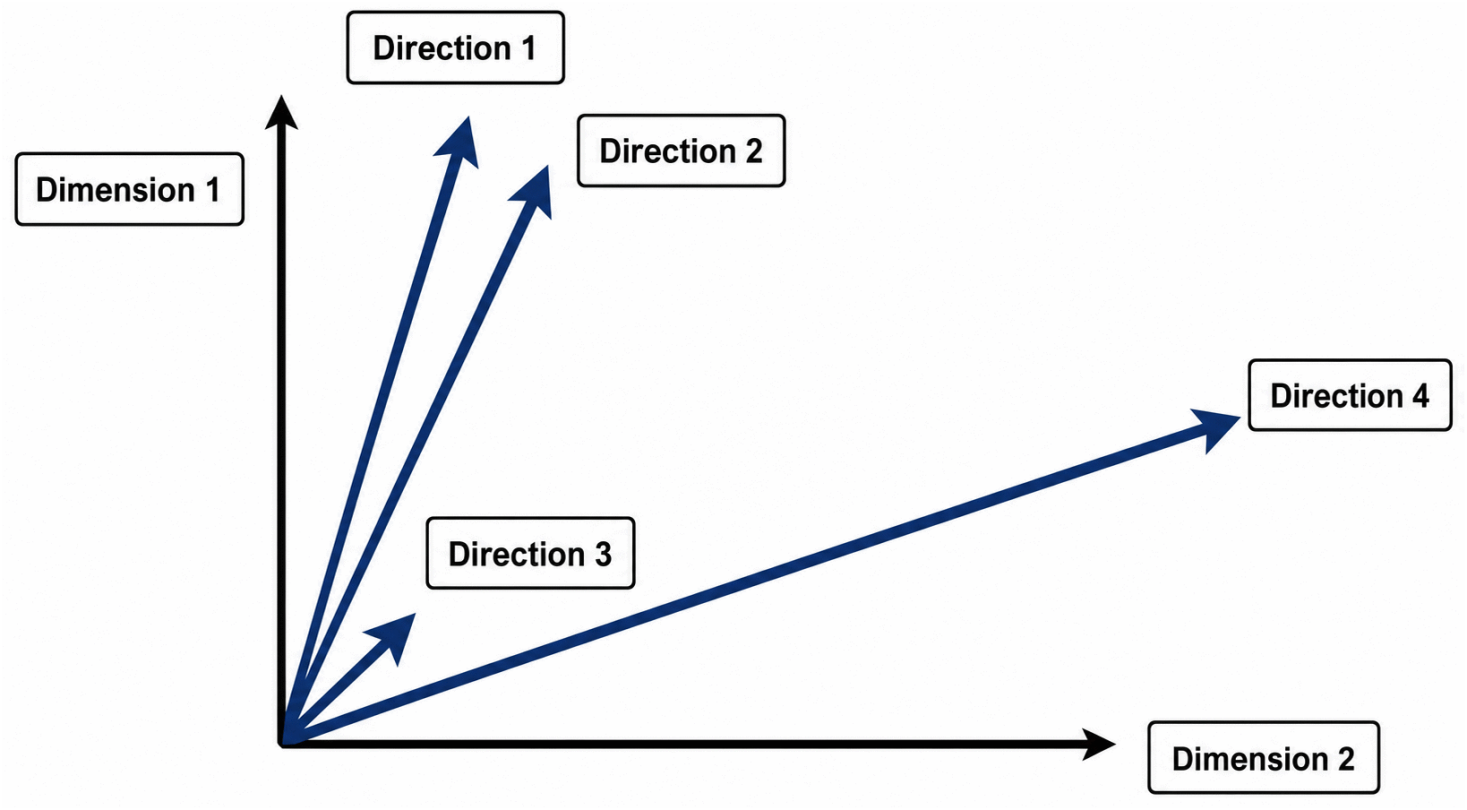
Dimensions and directions of vectors.

Furthermore, we implemented an iterative perturbation procedure [57], operating through combinations of directions in the 50-dimensional space for each latent vector. Fig 4 provides a schematic illustration for dimensions and directions in a two-dimensional distributional space with multiple potential directions. As seen in Fig 4, the moves in the four directions have different epsilons. In the context of our VAE model, however, the latent space was 50-dimensional rather than 2-dimensional, yielding a combinatorially vast, though technically finite, set of exploration directions. Therefore, the iterative approach was expected to enable a comprehensive traversal of the latent space, supporting diversity and stochasticity in the resulting outputs.

A concise summary of the perturbation-based XAI employed in this study is as follows: each test sample was subjected to an iterative perturbation procedure in the 50-dimensional latent space. Based on the assumption that multiple features collectively constitute style, we simultaneously perturbed eight dimensions (features) in each iteration. In other words, in each iteration, eight random unit dimensions were sampled, and the latent representation was nudged by a small amount (epsilon = 0.02) in the direction that maximally increased the probability of the target class, which referred to the opposite label from the original one. The search was performed for up to 100 iterations (as a heuristic upper limit) and was stopped early if a perturbation caused the predicted label to flip to the target class.

### Research questions

Two research questions (RQs) were proposed:

RQ1 Can the VAE-based classification model distinguish translated English from non-translated English?

RQ2 How can the DL model’s results be interpreted through the XAI method?

## Results and discussion

### The VAE-based model has distinguished the two text types

**Fig 5:**
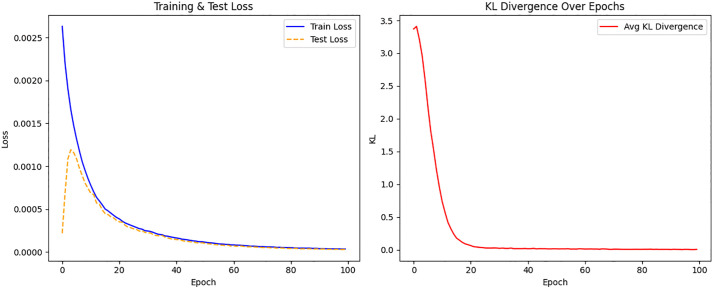
Training and test loss curves with KL Divergence of the VAE.

Fig 5 displays two metric curves from the VAE training over 100 epochs: the total loss, which measures the overall model error, and the KL divergence, which regularizes the latent space. As seen from the figure, the total loss has converged to a low value, and the KL divergence has stabilized, indicating that the model reconstructed data well and maintained a well-formed latent space.

Using the stacking method, we found that the best performance was achieved with a combination of the support vector machine, k-nearest neighbors, and the decision tree. As seen in Table 2, the analysis of logistic regression as the meta-model revealed that the support vector machine emerged as the most significant contributor to the ensemble with the highest coefficient score (3.79), followed by K-nearest neighbors and the decision tree, which also contributed but to a lesser extent.

**Table 2:** Coefficients of the best-performing base learners in the stacked ensemble.

Sub-model	Coefficient
Support Vector Machine	3.7935
K-nearest neighbors	0.9153
Decision Tree	0.6939

Table 3 summarizes the classification performance of the VAE-based model, using precision, recall, and F1-score, with support values indicating the number of samples in each class. Table 4 presents a five-fold cross-validation report of the mean, standard deviation (SD), and 95% confidence interval for each metric. The complete classification reports across five folds are provided in Appendix 1.

**Table 3:** Classification report of the VAE-based model.

	Precision	Recall	F1-score	Support
Non-translated English	0.94	0.81	0.87	96
Translated English	0.81	0.94	0.87	84
Accuracy	0.87			180
macro avg	0.88	0.88	0.87	180
weighted avg	0.88	0.87	0.87	180

**Table 4:** Classification performance variability across 5 folds.

Metric	Mean	SD Dev	95% Confidence Interval
Accuracy	0.8489	0.0203	[0.8207, 0.8771]
Recall	0.8502	0.0209	[0.8212, 0.8793]
F1-Score	0.8488	0.0203	[0.8206, 0.8769]

As shown in Table 3, the class support (96 vs. 84) indicates that the dataset was balanced. The model’s performance varied slightly between the two classes. For non-translated English, the model achieved a precision score of 0.94, a recall score of 0.81, and an F1-score of 0.87. For translated English, the model achieved a precision of 0.81, a recall of 0.94, and an F1-score of 0.87. Despite the minor differences in precision and recall, the model achieved an identical F1-score above 0.87 for both classes, indicating consistent overall performance across the two classes. The model’s total accuracy across 180 samples is 0.87, indicating it correctly classified 87% of instances. The macro average, which calculates the metric independently for each class and then takes the average, is above 0.87 for all three metrics. The weighted average, which weights each class’s score by its support, is also above 0.87 across all three metrics.

As for the classification performance variability, as shown in Table 4, the mean accuracy is 0.8489 with a SD of 0.0203, and a 95% confidence interval of [0.8207, 0.8771]. The mean recall is 0.8502 (SD = 0.0209) with a 95% confidence interval of [0.8212, 0.8793]. The mean F1-score is 0.8488 (SD = 0.0203), and the 95% confidence interval for F1-score is [0.8206, 0.8769]. These results indicate that the classification results are robust and stable, with all key metrics consistently falling within the narrow bounds of their respective confidence intervals across the five cross-validation folds.

**Fig 6:**
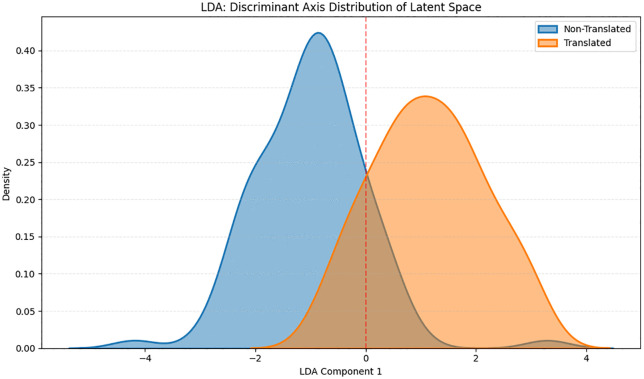
Linear Discriminant Analysis for the VAE’s latent space.

Fig 6 visualizes the distribution in the latent space through Linear Discriminant Analysis. Although there is some overlap in the middle region, indicating that a portion of the samples remains difficult to distinguish, the overall separation between the two classes along the discriminant axis is clear.

Table 5 and Table 6 show the performance of two baseline models: a logistic regression in the same latent space and a Term Frequency-Inverse Document Frequency (TF-IDF) model using 1–3 grams to incorporate n-grams as features.

**Table 5:** Classification report of logistic regression in the latent space.

	Precision	Recall	F1-score	Support
Non-translated English	0.73	0.75	0.74	96
Translated English	0.71	0.69	0.70	84
Accuracy	0.72			180
macro avg	0.72	0.72	0.72	180
weighted avg	0.72	0.72	0.72	180

**Table 6:** Classification report of a TF-IDF model (1-3 grams).

	Precision	Recall	F1-score	Support
Non-translated English	1.0	0.98	0.99	85
Translated English	0.99	1.0	0.99	95
Accuracy	0.99			180
macro avg	0.99	0.99	0.99	180
weighted avg	0.99	0.99	0.99	180

The utility of the VAE-derived latent space is supported by evaluating the two classes’ separability using logistic regression as a simple linear classifier. As seen in Table 5, despite its simplicity, the logistic regression model trained on the latent features achieved a clear, above-chance distinction (macro F1 = 0.72) between non-translated and translated English, indicating the latent space encoded meaningful learned representation relevant to the stylistic classification.

As seen in Table 6, the TF-IDF model achieved notably higher accuracy (macro F1 = 0.99), consistent with results from previous translation studies which used n-gram-based text classification for the same purpose [7,8,11]. However, the high accuracy was accompanied by a caveat as data sparsity (matrix sparsity = 0.8891). Due to the reliance on the Markov statistical assumption that each word depends only on preceding n-grams [61], data sparsity and overfitting pose a risk in the n-gram model, causing a problem known as the “zero-frequency problem ” [62–64].

In addition, as shown in Table 7, among the top 10 weighted distinguishing features of the TF-IDF model, most are function words, whose frequency differences offer mere insight into superficial stylistic tendencies [65] rather than deeper stylistic attributes. Besides functional words, named entity tokens, introduced through masking of geopolitical entities, frequently appear among the top features, primarily reflecting differences in geopolitical term frequencies across classes rather than conveying meaningful information about deeper stylistic patterns.

**Table 7:** Top 10 weighted features of the TF-IDF model.

Feature	Mean weight for Label 1	Mean weight for Label 2
The	0.3947	0.3012
Gpe	0.1116	0.1983
gpe gpe	0.0180	0.0826
We	0.0393	0.0854
gpe gpe gpe	0.0062	0.0436
To	0.1676	0.2034
Should	0.0402	0.0087
Loc	0.0492	0.0180
of the	0.0620	0.0321
Countries	0.0353	0.0069

In contrast, the value of the VAE’s representation learning cannot be judged by predictive scores alone. Instead, the VAE’s representation learning, i.e., learning a lower-dimensional, probabilistic representation, can support capturing broader distributed regularities and complex non-linear relationships [25,66], which are inherently missed by sparse, lexical-feature models like TF-IDF [67]. In this way, recurring discourse patterns can be encoded in a form that not only provides useful data for a downstream classification task but also offers a more flexible basis for a subsequent exploratory interpretation, e.g., the perturbation-based XAI for stylistic exploration.

In summary, with named-entity masking for geopolitical vocabulary and the removal of LDA-identified topical stop words to minimize topical confounds, the classification accuracy in the VAE-derived latent space consistently remained above 0.8. The VAE-based model also demonstrated stability across five-fold cross-validation and robustness with all accuracy scores exceeding 0.6 when a basic logistic regression classifier was used as the classifier. We thereby conclude that the VAE-based model effectively distinguished the two text types. Given that traditional n-gram models are prone to data sparsity, and considering that VAEs’ representation learning confers notable advantages, we contend that the latent space of VAEs offers a more flexible basis for exploratory stylistic analysis. We demonstrated this through the subsequent XAI-assisted analysis.

### Interpretations of stylistic differences go beyond lexical and syntactic features

In the perturbation tests, classification flips occurred with all the samples in the test dataset. Among the 23 misclassified samples, 18 non-translated English samples that had been misclassified as translated English were reclassified as non-translated and 5 translated English samples that had been misclassified as non-translated English were reclassified as translated English. Among the 131 correctly classified samples, 60 translated English samples were reclassified as non-translated English and 71 non-translated English samples were reclassified as translated English. Table 8 displays the descriptive statistics of the iterations of the perturbation-based XAI: the values ranged from 1 to 100 and there was a notable SD, indicating substantial variability in the number of iterations during the perturbation test. The distribution of the number of iterations was relatively dispersed (SD = 25). There were low values (1 time) and high values (100 times), reflecting considerable variability. The mean and median were close, suggesting that most samples had been perturbed around 40 times.

**Table 8:** The descriptives of the iterations for perturbations.

N	154
Missing	0
Mean	43.4
Media	40
Standard deviation	25
Minimum	1
Maximum	100

Table 9 shows the descriptive statistics of the cosine similarity used to match the decoded embeddings with texts from the original corpora. As shown in Table 9, across the 152 samples, the mean cosine similarity was 0.761 (SD = 0.0498), indicating reasonably close matches between the decoded embeddings and the embeddings of the retrieved texts.

**Table 9:** The descriptives of cosine similarity matching.

N	152
Missing	0
Mean	0.761
Media	0.763
Standard deviation	0.0498
Minimum	0.588
Maximum	0.822

On the basis of the XAI-assisted comparisons, we identified stylistic differences between translated and non-translated English beyond lexical and syntactic mismatch [9,38,39] and at a level of abstraction and complexity [12,16]. The stylistic differences were manifested as the emotional intensity in conveying opinions, expressing stances and making persuasive appeals, realized by a combination of lexical choices, syntactic patterns, tone selection and rhetorical devices. Specifically, non-translated English tended to be more emotionally expressive and use a wider range of rhetorical techniques. They included direct appeals, vivid descriptions, and strong sentence structures that create a sense of urgency and personal engagement. In contrast, translated English tended to be more neutral and reserved. It typically favored diplomatic language, cautious and formal wording, resulting in a less emotional and more detached tone. These differences remain clear in two types of texts discussing similar topics. We demonstrate our interpretations through the following two case examples. The complete perturbation results are provided in Appendix 2.

Case 1. A flip from non-translated to translated English

Example 1 is an excerpt from the translated English for Case 1.

“Hersh Goldberg-Polin loved geography…(the story of Goldberg-Polin). Devastation has a blurring effect… they relived the worst moments of their lives…Not only where he was, but who he was. Why his fate, and the fate of hundreds of hostages, mattered. And why the fate of thousands upon thousands of innocent Palestinians mattered, too. That they, too, were people, each containing a universe, who wanted and deserved peace, who wanted to and deserved to live. This past weekend, we learned the devastating news… Hersh, along with five other hostages, was brutally killed by Hamas…And so today, I want to speak to the many, many people living in agony…. And it reveals, yet again, the ugly truth about the vile…No member of this Council would tolerate their citizens being taken hostage and murdered. Not a single one of us. The United States strongly condemns Hamas’ brutality…Palestinian civilians are living in a hell on earth. Their lives continue to be put at risk by his cowardice and intransigence….”

Example 2 is an excerpt for Case 1 involving a decision flip to translated English.

“The situation in the Middle East requires an urgent diplomatic solution. For more than a year, we have seen devastating civilian suffering in Israel… And we have seen upheaval and unprecedented attacks…the United States has exercised leadership and resolve in pursuing clear objectives: End the war in Gaza…Avoid a broader regional war… And press for the full…These remain the United States’ objectives, and we do see a path to achieving them…Still, we will not give up on this diplomacy…. Israel must also urgently take additional steps to alleviate the catastrophic humanitarian situation in Gaza. The United States has been specific about what exactly Israel must do to… We are closely tracking Israel’s actions… and engaging with its leaders…Still, we need to see all steps fully implemented and sustained. And we need to see concrete improvement in the humanitarian situation on the ground. We need real and extended pauses …”

Both Example 1 and Example 2 address the ongoing Middle East crisis, specifically the violence and humanitarian suffering in Israel and Gaza in relation to Hamas. However, the two differ in rhetorical style and emotional tone. Example 1 seeks to persuade the audience through a personal narrative, invoking moral resonance and deep empathy. In contrast, Example 2 adopts a more diplomatic and rational approach, emphasizing explicit objectives and structured policies with “an urgent diplomatic solution”, “objectives” and “see a path to achieve them (objectives)”. While both examples contain emotionally charged language, Example 1 employs such expressions more frequently and with greater personal intensity, using phrases like “devastating”, “ugly truth”, “living in a hell”, “cowardice”, and “intransigence”. Additionally, Example 1 utilizes parallel sentence structures for emotional amplification. These sentence structures include “not only… but also”, “not a single”, and parallel patterns such as “who wanted…who wanted”. In contrast, the strongest language in Example 2 includes terms like “devastating”, “catastrophic”, “upheaval”, and “unprecedented”. These words are more concerned with describing circumstances than with conveying individual emotions.

Case 2. A flip from translated to non-translated English

Example 3 is the excerpt from the translated English in Case 2.

“The humanitarian situation in the country continues to worsen with 18 million people suffering from hunger and many parts of the country facing pronounced food insecurity…We call on all Sudanese parties... It is worth noting that funding shortfalls remain the biggest challenge.... The international community, traditional donors in particular, need to act responsibly by.... It must be pointed out that humanitarian relief efforts should... Humanitarian issues should not be politicized…. This is one of the major contributors to the prolonged turmoil in Sudan, which eventually plunged the country into a deep crisis. The tragedy unfolding in Sudan deserves in-depth reflection by the Council... A ceasefire and return to order in Sudan is the fundamental way to alleviate the humanitarian situation. We call on both parties to the conflict to implement Council Resolution 2724 by  .... The UN should step up coordination with the...”

Example 4 is the excerpt for Case 2 involving a decision flip to non-translated English

“The flames of war are still raging, and the humanitarian disaster is still intensifying. In the face of repeated violations... in the face of repeated breaches of the bottom... and in the face of enormous threats... The Security Council has no alternative but to immediately take further action...We must push for an immediate ceasefire with the utmost urgency. We must take pragmatic steps to alleviate the unprecedented humanitarian catastrophe. The World Health Organization Director-General has warned and described the conditions in Gaza as “hellish”. Security Council resolutions 2712 and 2720 must be fully implemented, and Israel must fully cooperate by removing...We must unswervingly promote the implementation of the two-State solution…Israel must immediately stop eroding the basis of the two-State solution, stop the forced displacement of the population of Gaza and the expansion of settlements in the West Bank, as well as the searches, arrests and attacks against Palestinians.  ...We call for an international peace...We support Palestine’s full membership… We have taken note of the accusations of the alleged involvement....”

Both Example 3 and Example 4 focus on the theme of ceasefire in conflict zones. Example 4 sounds more emotionally intense. In addition to the frequent use of “must”, the intensity is created by a parallel sentence structure “in the face of…in the face of… and in the face of…”, parallel phrases like “searches, arrests, and attacks” emphatic patterns such as “has no alternative but to…” and a rhetorical device in reference of flames and hell. In contrast, the focus of Example 3 is more on proposing diplomatic solutions to resolve the conflict rather than making impassioned appeals. Example 3 adopts a more restrained tone, favoring words like “should” and “need” over “must”. While it contains terms such as “prolonged turmoil” and “tragedy,” these were used primarily to describe the situation rather than to convey personal emotions.

In summary, drawing on the probabilistic representation learning of VAEs and perturbation-based XAI, we have interpreted stylistic differences between translated and non-translated English as global, subtle, and abstract properties that originate from, but extend beyond, the lexical and syntactic levels. The interpretations we have made demonstrates that our framework offers results’ interpretability in addition to classification accuracy. The utility of the framework supports our proposal that style emerges from the arbitrary and surface linguistic usages and VAEs capture style by transforming stochastic usages to organized probabilistic distributions in the latent. On the other hand, the value of the VAE’s latent space is more than providing data for classification as the downstream task; the latent space also provides an ideal explanatory basis for us to use XAI techniques to explore and interpret stylistic patterns and underlying regularities. By allowing for structured, interpretable perturbations within the latent space, VAE representations facilitate an examination of how style is encoded beyond what traditional models can offer.

## Conclusion

This study demonstrates an XAI DL framework for uncovering the distinctive stylistic features of translated languages. The framework integrates a BERT-based VAE for automated probabilistic feature extraction, a stacked ensemble-based majority vote for classification in the VAE’s latent space, and a perturbation-based XAI technique to facilitate result interpretations. Even after potential confounds that may risk conflating the classification accuracy were addressed, e.g., masking geopolitical vocabulary and removing topical signals unique to each corpus, the three classifiers still effectively distinguished between translated and non-translated English rendered at UN meetings. They achieved accuracy scores above 0.8 and made classification decisions consistent with two baseline models (a TF-IDF model and a logistic regression classifier in the latent space). In addition, the perturbation-based XAI method enabled an interpretation of the classification results beyond superficial lexical and syntactic features, extending our understanding of stylistic differences between translated and non-translated languages as global and subtle properties.

As a proof of concept, this study demonstrates that the three-stage framework balances adequate classification performance with the ability to interpret what the model has learned. It highlights the promise of the representation learning of DL models, such as VAEs’ probabilistic learning, in capturing complex stylistic traits for NLP classification tasks. It further highlights the value of a combination of DL models and XAI: DL models provide an explanatory basis for XAI, and integrating XAI is promising to result in interpretations and insights that go beyond mere predictive performance.

We acknowledge several limitations of our study. First, our findings are based on a single case study involving one language pair (Chinese-English) and a specialized corpus from UN meetings, which may narrow the generalizability of our results. Second, our workflow relies on a cosine similarity search to map decoded VAE embeddings back to discrete texts, rather than true text generation, which may limit the expressiveness and precision of reconstructed outputs. Third, there are other XAI methods, such as counterfactual analysis, that establish a firmer causal relationship between input and output to explain the model’s decisions. Finally, our work has not yet explored enhanced VAE architectures that might offer a richer or more fine-grained capture of stylistic differences.

Future research should extend this framework across diverse language pairs, discourse genres, and domains to assess its robustness and generalizability. Developing larger, more varied corpora and implementing advanced VAE variants, such as hierarchical or style-augmented VAE, could yield deeper insights. Improvements in text generation methods for decoding VAE latent vectors may enable more precise and faithful retrieval of stylistic elements. Finally, future studies could cross-validate XAI methods to further elucidate and interpret the decision-making processes of the DL models.

## Supporting information

S1 File Classification reports across five folds. (TXT)

S2 File The perturbation results. (XLSX)
